# Reduction of 3-Deoxyglucosone by Epigallocatechin Gallate Results Partially from an Addition Reaction: The Possible Mechanism of Decreased 5-Hydroxymethylfurfural in Epigallocatechin Gallate-Treated Black Garlic

**DOI:** 10.3390/molecules26164746

**Published:** 2021-08-05

**Authors:** An-Ting Tu, Jer-An Lin, Chieh-Hsiu Lee, Yi-An Chen, Jung-Tsung Wu, Ming-Shiun Tsai, Kuan-Chen Cheng, Chang-Wei Hsieh

**Affiliations:** 1 Department of Food Science and Biotechnology, National Chung Hsing University, 145 Xingda Rd., South Dist., Taichung 402, Taiwan; ballann3421126@gmail.com (A.-T.T.); a0926449030@gmail.com (C.-H.L.); 2 Graduate Institute of Food Safety, National Chung Hsing University, 145 Xingda Rd., South Dist., Taichung 402, Taiwan; lja@dragon.nchu.edu.tw; 3 College of Biotechnology and Bioresources, Da-Yeh University, 168 University Rd., Dacun, Chang-Hua 515, Taiwan; anncerita@gmail.com (Y.-A.C.); tony@futis.com.tw (J.-T.W.); 4 Department of Food Science and Biotechnology, Da-Yeh University, 168 University Rd., Dacun, Chang-Hua 515, Taiwan; tsaims1@mail.dyu.edu.tw; 5 Graduate Institute of Food Science and Technology, National Taiwan University, 1, Sec 4, Roosevelt Rd., Taipei 106, Taiwan; kccheng@ntu.edu.tw; 6 Institute of Biotechnology, National Taiwan University, 1, Sec 4, Roosevelt Rd., Taipei 106, Taiwan; 7 Department of Medical Research, China Medical University Hospital, Taichung 404, Taiwan; 8 Department of Optometry, Asia University, 500 Lioufeng Rd., Wufeng, Taichung 404, Taiwan

**Keywords:** black garlic, 5-Hydroxymethylfurfural, 3-deoxyglucosone, epigallocatechin gallate, LC-MS/MS

## Abstract

5-Hydroxymethylfurfural (5-HMF) is a harmful substance generated during the processing of black garlic. Our previous research demonstrated that impregnation of black garlic with epigallocatechin gallate (EGCG) could reduce the formation of 5-HMF. However, there is still a lack of relevant research on the mechanism and structural identification of EGCG inhibiting the production of 5-HMF. In this study, an intermediate product of 5-HMF, 3-deoxyglucosone (3-DG), was found to be decreased in black garlic during the aging process, and impregnation with EGCG for 24 h further reduced the formation of 3-DG by approximately 60% in black garlic compared with that in the untreated control. The aging-mimicking reaction system of 3-DG + EGCG was employed to determine whether the reduction of 3-DG was the underlying mechanism of decreased 5-HMF formation in EGCG-treated black garlic. The results showed that EGCG accelerated the decrease of 3-DG and further attenuated 5-HMF formation, which may be caused by an additional reaction with 3-DG, as evidenced by LC-MS/MS analysis. In conclusion, this study provides new insights regarding the role of EGCG in blocking 5-HMF formation.

## 1. Introduction

Black garlic is produced from white garlic at high temperature under controlled humidity. The color of the garlic turns black, and the garlic develops a sweet flavor because of the Maillard reaction that occurs during processing [1]. Additionally, the characteristic pungent and spicy odor of raw garlic is omitted. The aging of garlic can increase the levels of functional compounds, but it is also accompanied by the production of the harmful substance 5-Hydroxymethylfurfural (5-HMF). 5-HMF has been confirmed to have indirect mutagenicity [2], genotoxicity [3], carcinogenicity [4] and other negative effects. Lee et al. [5] previously discussed some strategies to reduce 5-HMG formation, including ultraviolet irradiation [6], addition of flavanols [7,8], yeast fermentation [9], vacuum treatment [10], microwave heating [11], nonthermal processing [12], formulation adjustment [13] and encapsulation [14]. These strategies have been shown to reduce 5-HMF formation by more than 20%. The mechanisms underlying these strategies include 5-HMF degradation and formation of adducts or reduction of Maillard reaction matrix activity to inhibit the formation of 5-HMF. 

**Figure 1:**
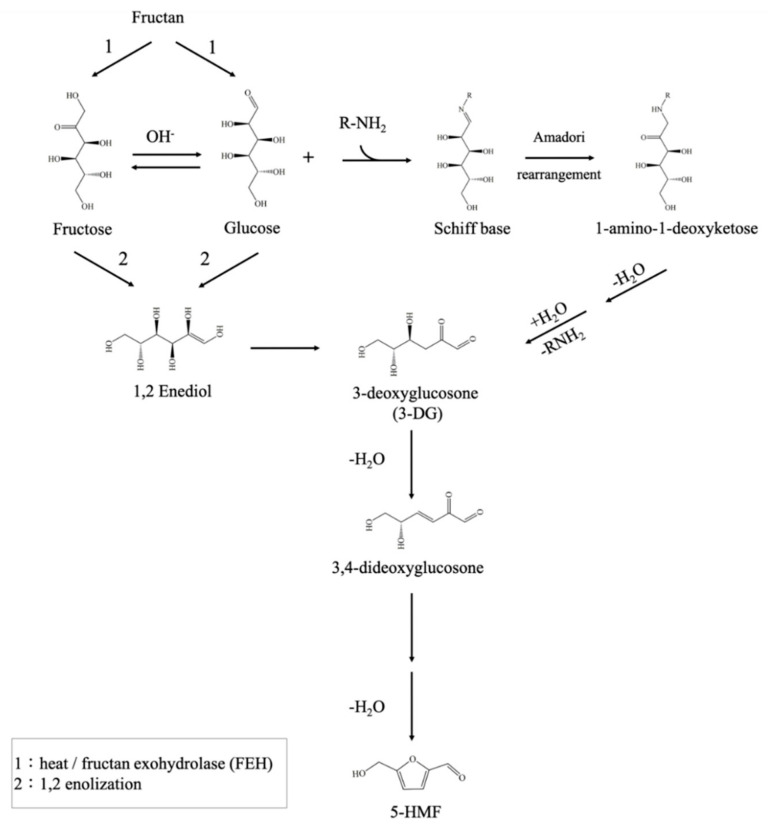
Mechanism of 5-HMF formation in black garlic [5,8,16].

Fructan is the main polysaccharide in garlic. During the aging period, fructan can be degraded under thermal treatment and catalyzed by fructan exohydrolase into glucose and fructose [15], which in turn generate 5-HMF via caramelization and the Maillard reaction (Figure 1). 5-HMF is primarily formed by the Maillard reaction and sugar dehydration under acidic conditions [5,16]. When subjected to heat, glucose reacts with amino acids to form a Schiff base that is cyclized to further form Amadori rearrangement products. Because Amadori rearrangement products are not stable, they are transformed into 1,2-eneaminnol and 3-DG [8,16,17], which is then dehydrated to 3,4-dideoxyglucosone and cyclized to finally yield 5-HMF. The generation of 3-DG is thought to be a key intermediate product of this reaction [8,16]. Although fructose can also form 5-HMF under acid catalysis, the literature indicates that the fructofuranosyl cation can be easily converted into 5-HMF in relatively dry systems [18]. In a high-humidity environment, 5-HMF in black garlic is unlikely to be formed through the dehydration of fructofuranosyl cation. In addition, glucose and fructose can also be dehydrated and cyclized to form 5-HMF after 1,2-enolization, further dehydrating to form 3-DG alone [16].

In addition to being a key intermediate product of 5-HMF, 3-DG is also regarded as an indicator of the aging of beer and wine, which can affect the final sensory quality of beer and wine [19,20]. In addition, 3-DG has also been reported to cause changes in food texture, flavor and taste during food processing [21]. However, a highly reactive sugar and carbonyl intermediate, 3-DG is notorious for binding with proteins to create advanced glycation end-products (AGEs), which promote the development of numerous chronic and degenerative diseases, such as cancer, diabetes, hypertension and atherosclerosis [13,22,23]. Currently, efforts by the food industry to prevent or minimize 3-DG formation during production include alkali treatment as well as the addition of endogenous enzymes and polyphenols in the ingredient mixtures [13,24,25,26]. The role of polyphenols in 5-HMF formation has attracted much attention in recent years, especially flavan-3-ols. Flavan-3-ols have the ability to adduct with various carbonyl intermediates due to their nucleophilic sites (C6 and C8) on the A ring [7], leading to a reduction in the overall 5-HMF content in foods [8].

Inhibition of 5-HMF formation using flavanol compounds has previously attracted much attention. A report indicated that quercetin can be combined with 5-HMF and its precursor 3,4-dideoxyglucosone-3-ene (3,4-DGE), to reduce the formation of 5-HMF in bread by 86% [8]. Qi et al. [7] reported that the mechanism of flavanols for reducing 5-HMF formation might be related to their ability to capture carbonyl groups. The C6 and C8 of flavanols are nucleophilic and can, thus, react with active carbonyl intermediates and 5-HMF, thereby inhibiting and reducing the formation of 5-HMF by 50%. Our research team previously confirmed that impregnating epigallocatechin gallate (EGCG) can effectively reduce the formation of 5-HMF in black garlic. During the 30-day aging period, the 5-HMF content of garlic with EGCG impregnation for 24 h was 2.16 ± 0.12 mg/g dry weight, representing a 55% reduction in the formation of 5-HMF relative to that in the control [27]. Nevertheless, the mechanism of 5-HMF reduction is still not understood. Based on previous studies, the present study aims to use a model and high-performance liquid chromatography-tandem mass spectrometry (HPLC-MS/MS) analysis to confirm how EGCG affects HMF formation by an inhibitory mechanism.

## 2. Results and Discussion

### 2.1. Effect of Various Impregnation Conditions on 3-DG Content during the Aging Process

**Figure 2:**
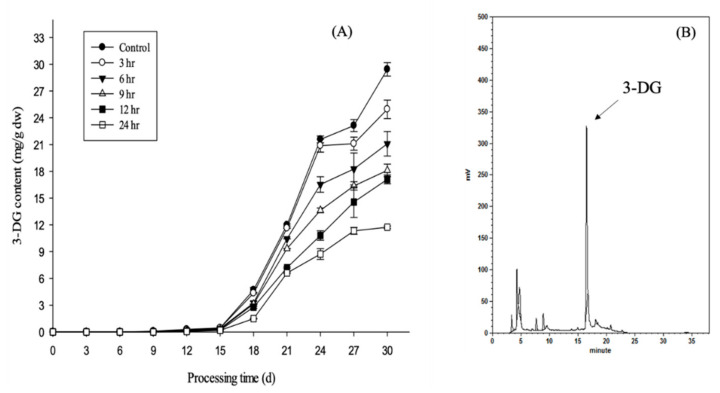
(**A**) 3-DG content (mg/g dry weight) in garlic with different impregnation times (0, 3, 6, 9, 12 and 24 h) during aging for 30 days. All values are means ± standard deviation (*n* = 3). (**B**) HPLC chromatogram of black garlic at 312 nm.

The changes in 3-DG content in garlic aged for 30 days with or without EGCG impregnation are presented in Figure 2. 3-DG was first observed on the 9th day, although its content did not increase rapidly until after the 15th day. Samples impregnated with EGCG displayed significantly lower levels of 3-DG than the controls, and on the 30th day, the 3-DG contents (mg/g dry weight) after 0, 3, 6, 9, 12 and 24 h of EGCG impregnation were 29.43 ± 0.75, 24.95 ± 1.03, 21.10 ± 1.37, 18.11 ± 0.70, 17.10 ± 0.49 and 11.73 ± 0.15, respectively. Garlic with EGCG impregnation for 24 h had 60% less 3-DG content than the control. This result demonstrates that the formation of 3-DG during the aging process is inversely correlated with the duration of EGCG exposure, and EGCG can react with 3-DG to reduce the content of 3-DG during the aging of black garlic.

EGCG impregnation can reduce 5-HMF formation during the aging process of black garlic, as shown in our previous studies. As the impregnation time increases (from 3 h to 24 h), the 5-HMF content subsequently decreases. The 5-HMF content of garlic with EGCG impregnation for 0, 3, 6, 9, 12 and 24 h at 30 days of aging was 4.79 ± 0.03, 4.41 ± 0.07, 3.89 ± 0.10, 3.30 ± 0.17, 2.37 ± 0.11 and 2.16 ± 0.12 mg/g dry weight, respectively. Among different impregnation times, the 5-HMF content of garlic impregnated with EGCG for 24 h was 2.16 ± 0.12 mg/g dry weight, which reduced the formation of 5-HMF by approximately 55% compared with that in the control [27]. Many studies have used different processing technologies to reduce the 3-DG content in food. In fermented milk, the contents of 3-DG and 5-HMF are reduced by hydrolyzing endogenous lactose. In addition to maintaining the color of the product without additional sugar, the technique is also suitable for products marketed to people with lactose intolerance [13]. Moreover, coffee beans undergo alkali treatment before roasting, which can also effectively reduce the content of 3-DG and 5-HMF [26]. In a study by Kokkinidou an Peterson [24], polyphenols such as catechins were also used to reduce the content of glyoxal, methylglyoxal and 3-DG in ultra-high temperature-treated milk. EGCG is the class of flavanols. Qi et al. [7] indicated that the C6 and C8 sites of flavanols are nucleophilic and react with reactive carbonyl intermediates to reduce their content. Epicatechin can react with 3-DG to inhibit the formation of Maillad reaction products [28]. The use of processing technologies to reduce the formation of Maillard reaction products in food that may be harmful to human health has become an important food safety issue and received significant attention in recent years.

### 2.2. Kinetic Study of Various Impregnation Conditions on 3-DG Content during Aging of Garlic

The rate constant (*k*) and R^2^ values for the zero- and first-order equations of 3-DG formation in black garlic after EGCG impregnation are presented in Table 1. Under the zero-order reaction, *k*, pertaining to 3-DG formation, decreased with increasing EGCG impregnation time. Additionally, k was 1.5514, 1.3792, 1.1717, 1.0075, 0.9029 and 0.6716 at EGCG impregnation times of 0, 3, 6, 9, 12 and 24 h, respectively. This reaction also had a higher R^2^ value, indicating that the EGCG impregnation data for 3-DG formation as a function of time were better fitted using a zero-order equation.

Arena, Ballistreri and Fallico [29] also used a zero-order kinetic equation to describe the changes in the 3-DG and 5-HMF contents when honey was stored at different temperatures; they found a good correlation between 3-DG and 5-HMF when honey was stored at different temperatures. These findings are in accordance with the results of Lee et al. [27]. Zhang et al. [30] investigated different heating times and temperatures on the formation of α-dicarbonyl compounds in the thermal reaction model. The formation of 3-DG was well fitted by zero-order kinetics, and the amount of 3-DG increased linearly with heating time. This result is consistent with previous results showing that the 3-DG content increased with heating time, and the formation of 3-DG followed zero-order kinetics.

### 2.3. Effects of EGCG on 3-DG and 5-HMF Content in an Aging-Mimicking Model System

**Figure 3:**
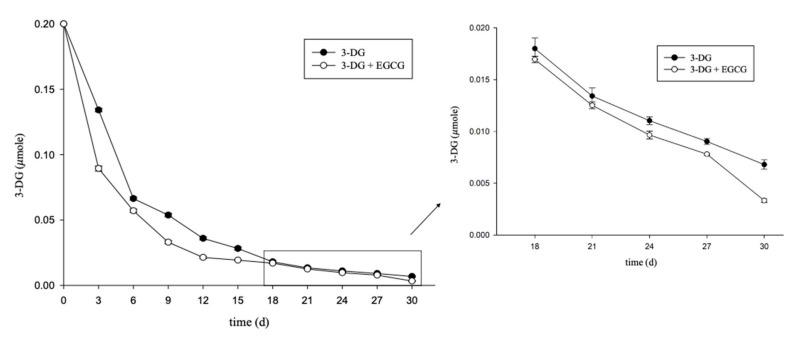
Kinetics curves of 3-DG in the 3-DG or 3-DG + EGCG model heated at 70 °C for 30 days.

**Figure 4:**
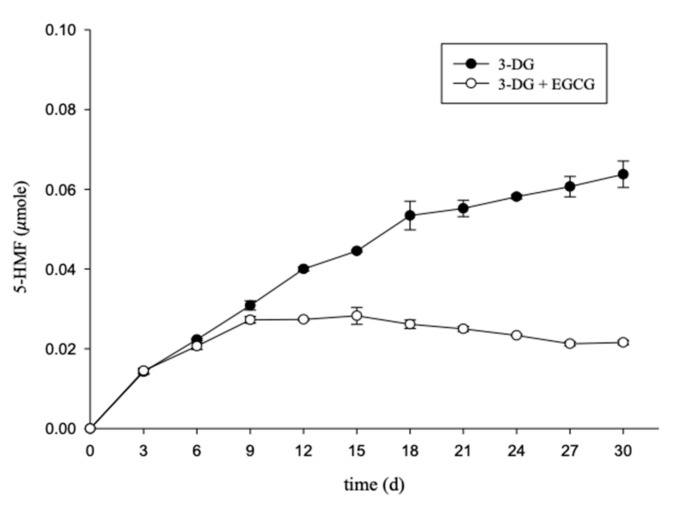
Kinetics curves 5-HMF in the 3-DG or 3-DG + EGCG model heated at 70 °C for 30 days.

Figure 3 shows that the group that was impregnated with EGCG displayed a faster reduction in 3-DG content during the early stage, and the 3-DG content in both groups was lower, whereas the change tended to be stable in the later stages of thermal treatment. These results suggest that EGCG reacts with 3-DG, resulting in a reduction of its content. Figure 4 shows that after the 9th day of heat treatment, the 5-HMF content in the group that did not receive EGCG impregnation was higher than that in the EGCG treatment group. However, the change in 5-HMF content in the group with EGCG treatment tended to be constant. Importantly, on the 30th day, the 5-HMF content in the group treated with EGCG was 66% that in the control. In the early stage of heat treatment, the 3-DG content may decrease because of the adducts of 3-DG and EGCG. In the later stages of heat treatment, the 5-HMF content may decrease due to the adducts of 3-DG and EGCG, which can reduce the 3-DG and 5-HMF contents, thereby reducing 5-HMF production. Previous studies showed that adding EGCG in high-fructose corn syrup and storing it at 35 °C can reduce the content of 3-DG, MGO and 5-HMF compared with those in the control. EGCG can be regarded as a polyphenol with the potential of reducing the content of active carbonyl compounds in beverages [25].

### 2.4. Identification of the Adducts from the Reaction of EGCG and 3-DG in an Aging-Mimicking Model System

**Figure 5:**
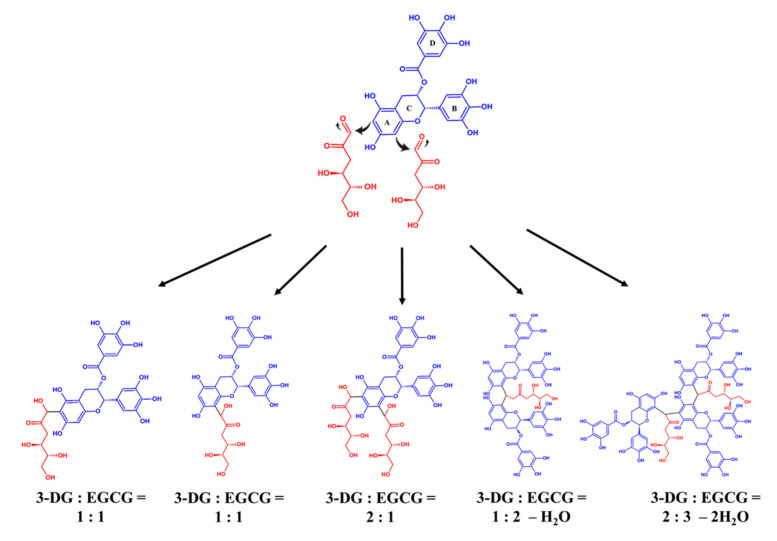
Possible formation pathway of adducts from the reaction of 3-DG and EGCG (EGCG: blue; 3-DG: red).

Figure 5 shows the possible formation pathway for adducts of 3-DG and EGCG. Sang et al. [31] reported that methylglyoxal (MGO) and EGCG can form adducts at a ratio of 1:1 and 2:1. The results also demonstrated that the C6 and C8 positions in the A-ring of EGCG are the two active sites for trapping reactive dicarbonyl compounds and forming adducts. Totlani and Peterson [32] showed that epicatechin in the Maillard reaction model could form bonds with reactive dicarbonyl compounds to reduce the content of dicarbonyl compounds. EGCG has a similar trapping mechanism and can form adducts with reactive dicarbonyl in the model reaction system [32,33]. MGO and 3-DG are dicarbonyl compounds, so it was speculated that 3-DG also forms adducts with EGCG at a ratio of 1:1 and 2:1. 3-DG is a key intermediate product in the formation of 5-HMF [16]. Previously, Qi et al. [7] indicated that epicatechin can form adducts through a dehydration reaction with 5-HMF at a ratio of 2:1 and 3:2 via its nucleophilic sites (C6 and C8). Considering the fact that epicatechin and EGCG are molecularly related, it was hypothesized that EGCG would also form an adduct with 3-DG at a similar ratio. Since our previous study proved that EGCG impregnation could reduce 5-HMF formation effectively during the aging of black garlic, we speculated that EGCG and 3-DG could form adducts through a dehydration reaction at a ratio of 2:1 and 3:2, which further attenuate 5-HMF formation.

**Figure 6:**
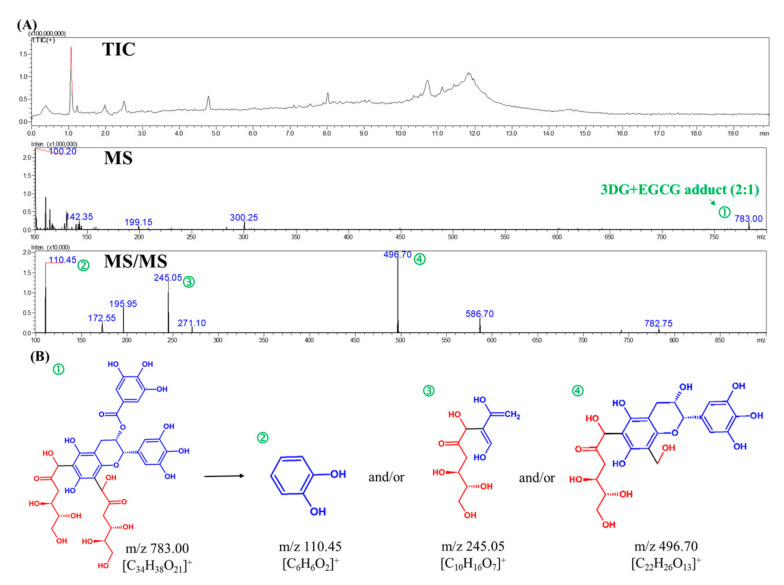
(**A**) Total ion chromatogram, MS spectra and MS/MS spectra of a molecule with an *m*/*z* of 783 for products generated from the reaction of EGCG and 3-DG at 70 °C for 30 days. (**B**) Scheme of the fragmentation of the 3-DG–EGCG adducts observed in MS/MS analysis (EGCG: blue; 3-DG: red). TIC: total ion chromatogram.

The adduct in the model of 3-DG and EGCG was identified using HPLC-MS/MS. The structure, extract ion chromatogram and mass spectrum of the adduct are shown in Figure 6. In the positive ion mode, the molecular ion peak with *m*/*z* 783 ([M + H]^+^) matched with the adducts composed of EGCG and two 3-DG. Fragment 496.70 ([M − 286 + H]^+^) represents the loss of the pyrogallol group and 3-DG, forming the A, B and C rings of EGCG and 3-DG. Chen et al. [34] showed that in the adduct of catechin and methylglyoxal (*m*/*z* 433), the fragment ion at *m*/*z* 361 represents the loss of methylglyoxal. A similar result was also obtained for epicatechin–methylglyoxal adducts. A fragment with an *m*/*z* of 245.05 ([M–539 + H]^+^) is produced by the loss of 4-(4-hydroxy-3,5-dimethoxybenzoyl)oxy-3,5-dimethoxybenzoic acid and 3-DG. A fragment with an m/z of 110.45 ([M–674 + H]^+^) is produced by the loss of (5,7-dihydroxychroman-3-yl) 3,4,5-trihydroxybenzoate and 3-DG. Our results show that the adduct of EGCG-3-DG was formed in the model, as studied using HPLC-MS/MS analysis. 3-DG could form adduct with EGCG in a ratio of 2:1 and bind to EGCG at C6 and C8 of the A-ring, which is consistent with previous studies [31,34]. EGCG showed an inhibition effect on the formation of browning precursors through forming adducts with Amadori rearrangement products, which further inhibit the Maillard reaction [35]. Furthermore, the results are in accordance with those presented in Figure 3 and Figure 4. In the later periods of heat treatment, 3-DG formed adducts with EGCG to reduce the 3-DG content. The change in 5-HMF content in the model tended to be stable. Thus, heating at 70 °C for 30 days will cause the formation of the adduct of 3-DG and EGCG in the model, which further reduce the formation of 5-HMF.

## 3. Materials and Methods

### 3.1. Materials and Sample Preparation

Garlic (*Allium sativum*) was grown in Cihtong Township, Yunlin County, Taiwan, and provided by Ajins Biomedical Corporation. Fresh garlic bulbs were impregnated with 5% EGCG solution at 40 °C for 3, 6, 9, 12 or 24 h and then heated at 70 °C with 80% relative humidity for 30 days [1]. All chemicals and solvents were of analytical grade.

### 3.2. Measurement of 3-DG and 5-HMF Content

The samples were mixed with double-distilled water and subjected to ultrasound for 30 min. The supernatant was then mixed with 0.2% ortho-phenylenediamine, incubated in a dark room for 12 h and then filtered through 0.22-μm syringe filters. Finally, this mixture was injected into a high-performance liquid chromatography (HPLC) instrument equipped with a photodiode array detector (L-2455, Hitachi, Tokyo, Japan). The 3-DG content was measured according to previous study [29]. Acetic acid and methanol were used as the mobile phases with a flow rate of 0.7 mL/min. The injection volume was 20 μL. The absorbance of the eluent at 312 nm was recorded. The 5-HMF content was measured according to a previous study [36]. HPLC coupled to a photodiode array detector. Distilled water and acetonitrile (88:12, *v*/*v*) were used as the mobile phases with a flow rate of 1.0 mL/min. The injection volume was 20 μL. The absorbance of the eluent at 284 nm was recorded.

### 3.3. Kinetic Study

The zero-order and first-order Equations (1) and (2) were used to evaluate the reaction order:

(1) $$C-C_{0}\;=\;k\times t$$

(2) $$ln\left(C/C_{0}\right)\;=\;k\times t$$

where *C* represents the 3-DG content (mg/g dry weight) at specific time points, *C*_0_ represents the 3-DG content (mg/g dry weight) at time 0, *k* is the rate constant (mg g^−1^ day^−1^) and *t* represents the processing time [6].

### 3.4. Identification of Adducts from the Reaction of EGCG and 3-DG in the Aging-Mimicking Model Using HPLC-MS/MS

To determine whether EGCG can reduce the formation of 5-HMF by combining 3-DG, an aging-mimicking model was developed and analyzed. In this experiment, 1 mL of 3-DG (0.2 mM) and 1 mL of EGCG (0.8 mM) were mixed and placed at 70 °C with 80% relative humidity for 30 days and then analyzed using LC-MS/MS (Shimadzu LC-MS-8045 triple quadrupole mass spectrometer) with slight modifications [37]. Chromatographic separation was achieved using a C18 column (1.9 μm, 2.1 mm × 100 mm, Agela, Tianjin, China) with a flow rate of 1 mL/min. The mobile phase consisted of acetonitrile (solvent A) and 0.1% formic acid aqueous solution (solvent B). The gradients of elution were as follows: 0–5 min, 98–80% B; 5–8 min, 80–60% B; 8–10 min, 60–20% B; and 10–12 min, 20–98% B. Mass spectrometry was performed in positive electrospray ionization (ESI+) mode. The instrumental parameters of electrospray mass spectrometry were as follows: scan speed, 5000 amu/s; nebulizing gas flow, 3 L/min; heating gas flow, 10 L/min; interface temperature, 300 °C; heat block temperature, 400 °C; and drying gas flow, 10 L/min. AB SCIEX PeakView™ software 2.2 was used for processing and interpreting accurate mass data.

### 3.5. Statistical Analysis

All data were expressed as means ± standard deviations. The data were evaluated using one-way analysis of variance in IBM SPSS Statistics 20. Significant differences were determined using Duncan’s multiple range test at *p* < 0.05 [38].

## 4. Conclusions

In this study, we demonstrated that black garlic impregnated with EGCG had 60% less 3-DG than the control. Kinetics studies have shown that EGCG will stably combine with 3-DG during the heat treatment, and then reduce the content of 3-DG. The result of LC-MS/MS further proved that EGCG would combine with the reactive dicarbonyl compounds of 3-DG, which delays the generation rate of 5-HMF 66% on 30 days of heat treatment.

Consequently, EGCG impregnation in black garlic may be a promising and novel strategy for mitigating the development of harmful intermediates, including 3-DG and 5-HMF, formed during the Maillard reaction. It is the first study to explore using EGCG to decrease 5-HMF formation through lower 3-DG content. In future research, it may provide the application on other thermal processing foods to maintain products quality. Reducing the formation of 5-HMF effectively can lower human dietary intake and its negative effects. The finding of this study provides value for potential industrial development.

## Figures and Tables

**Table 1:** Values of k and R^2^ for zero-order and first-order equations of 3-DG formation in garlic with or without EGCG impregnation.

Group	Zero-Order Equation		First-Order Equation	
	*k*	R^2^	*k*	R^2^
Control	1.5514	0.9217	0.3014	0.9095
3 h	1.3792	0.9160	0.3466	0.8689
6 h	1.1717	0.9189	0.3727	0.8482
9 h	1.0075	0.9288	0.3090	0.8979
12 h	0.9029	0.9308	0.3028	0.8600
24 h	0.6716	0.9127	0.3180	0.8514

## Data Availability

All data related to this study are presented in this publication.

## Sample Availability

Samples are not available from the author.
